# When Parasites Are Good for Health: Cestode Parasitism Increases Resistance to Arsenic in Brine Shrimps

**DOI:** 10.1371/journal.ppat.1005459

**Published:** 2016-03-03

**Authors:** Marta I. Sánchez, Inès Pons, Mónica Martínez-Haro, Mark A. Taggart, Thomas Lenormand, Andy J. Green

**Affiliations:** 1 Department of Wetland Ecology, Estación Biológica de Doñana, (EBD-CSIC), Seville, Spain; 2 Department of Life Sciences, Marine and Environmental Sciences Centre (MARE), University of Coimbra, Coimbra, Portugal; 3 Instituto de Investigación en Recursos Cinegéticos (IREC-CSIC-UCLM-JCCM), Ciudad Real, Spain; 4 Environmental Contamination and Ecological Health, Environmental Research Institute, University of the Highlands and Islands, Thurso, Scotland, United Kingdom; 5 Department of Genetic and Evolutive Ecology, Center of Functional Ecology and Evolution (CEFE), National Centre for Scientific Research (CNRS), Montpellier, France; Universidade de Aveiro, PORTUGAL

## Abstract

Parasites and pollutants can both affect any living organism, and their interactions can be very important. To date, repeated studies have found that parasites and heavy metals or metalloids both have important negative effects on the health of animals, often in a synergistic manner. Here, we show for the first time that parasites can increase host resistance to metalloid arsenic, focusing on a clonal population of brine shrimp from the contaminated Odiel and Tinto estuary in SW Spain. We studied the effect of cestodes on the response of *Artemia* to arsenic (acute toxicity tests, 24h LC_50_) and found that infection consistently reduced mortality across a range of arsenic concentrations. An increase from 25°C to 29°C, simulating the change in mean temperature expected under climate change, increased arsenic toxicity, but the benefits of infection persisted. Infected individuals showed higher levels of catalase and glutathione reductase activity, antioxidant enzymes with a very important role in the protection against oxidative stress. Levels of TBARS were unaffected by parasites, suggesting that infection is not associated with oxidative damage. Moreover, infected *Artemia* had a higher number of carotenoid-rich lipid droplets which may also protect the host through the “survival of the fattest” principle and the antioxidant potential of carotenoids. This study illustrates the need to consider the multi-stress context (contaminants and temperature increase) in which host-parasite interactions occur.

## Author Summary

Virtually all free-living organisms are infected by parasites. Moreover, both parasites and hosts may be exposed to increasing levels of pollution and might be affected by climate change. However, few studies have considered the environmental context in which parasites and hosts interact, and the relationships between these factors remains poorly understood. It is assumed that infection with parasites increases mortality under a cause of stress such as pollution. We studied the combined effect of arsenic (As) pollution, temperature increase and infection by tapeworms on the health of the economically and ecologically important brine shrimp *Artemia*. We found that tapeworms make *Artemia* ***more*** resistant to As, a major pollutant in aquatic environments, even under increased temperature conditions. These parasites increase the capacity of antioxidant enzymatic defenses, allowing infected individuals to cope better with As. Moreover, tapeworms increase fat reserves in their hosts, which may be advantageous due to the ability of lipids to sequester pollutants (“survival of the fattest” principle). Although our results may be unusual, we find a clear explanation for them. This makes them of broad significance and general interest.

## Introduction

Many aspects of host-parasite interactions have been studied in detail, from molecular mechanisms to adaptive strategies and their ecological and evolutionary consequences (reviewed in Schmid-Hempel 2011 [1]). In contrast, few studies have considered the context of multiple environmental stressors in which host-parasite interactions occur in natural conditions. Consequently there are important limitations to our understanding of the ecology and evolution of host-parasite interactions, and to our ability to reach reliable conclusions. For example, increasing numbers of taxa (both, free living and parasites) are exposed to pollution and impacted by climate change [2]. However, the complex relationships between these factors (i.e. parasites, pollution and climate change) are not well understood.

Parasites and hosts can react differently to pollutants, influencing their mutual interactions. For instance, if the parasite is more susceptible than the host to the pollutant, the exposure to pollution provides the indirect benefit (for the host) of protecting against the parasite. Conversely, the epidemiology of the parasite may be altered negatively if the pollutant impacts the life history of the host (e.g. reducing survival), thus compromising parasite transmission. Moreover, parasites and pollution can interact to affect the health of the host, the central topic of the emerging field of “Environmental Parasitology” [3]. Most studies evaluating the joint effect of parasites and pollution on the health of free-living organisms find that there are additive or synergistic effects between these stressors [4,5,6]. For example, coinfection of amphipods by acanthocephalans and microsporidians led to a reduction in antitoxic defenses when exposed to cadmium [7].

However, parasites can influence multiple facets of host phenotype [8], including physiology, behavior and biochemistry, so they may change the host response to a pollutant in diverse and complex ways. The physiology of the parasite itself may have a direct influence, e.g. through the capacity observed in several parasite groups to bioaccumulate contaminants [9,10]. Therefore, more studies are required to understand the diversity of ways parasites and pollution can interact to affect the health of organisms. Of particular concern are the mechanistic (proximate) bases of host-parasite interactions; for example the potential of oxygen-free radicals and other reactive oxygen species (ROS) to induce oxidative damage in tissues and cellular components, leading to adverse health effects and diseases. The evaluation of the levels of antioxidant enzymes in infected and uninfected organisms can provide valuable information on antioxidant status and their capacity to confront multiple stress conditions.

Furthermore, all these effects are likely to depend on physiological variation which is influenced by temperature and hence by climate change. The projected temperature increase this century [11] is likely to increase the toxicity of pollutants [12] as well as the transmission, distribution and abundance of many parasites [13,14,15]. To date, studies of interactions between parasites and pollutants on hosts have only been carried out at a single temperature, with the exception of one investigation [16] dealing with the effect of trematode infection and seasonal temperature on bioaccumulation of xenobiotics in freshwater clams.

Brine shrimps *Artemia* spp. (Branchiopoda, Anostraca) are economically and ecologically important. They are used as model organisms in aquatic toxicology [17, 18] and are the most important live food used in aquaculture worldwide [19]. *Artemia* are the dominant macroinvertebrate in hypersaline ecosystems around the world, and many waterbirds depend on them as food [20,21,22]. They control phytoplankton populations [23] and are also intermediate hosts for a rich community of avian cestodes [24]. These parasites cause strong physiological and behavioural changes in *Artemia* [25,26,27] which may be expected to influence their response to pollutants.

We studied the *Artemia parthenogenetica* population from the Odiel and Tinto estuary, SW Spain, one of the most polluted estuarine systems in Western Europe [28]. *A*. *parthenogenetica* from Odiel have high levels of cestode infection [29], especially of *Flamingolepis liguloides* which uses flamingos as final hosts, and *Confluaria podicipina* which infects grebes [30]. Arsenic is a highly toxic and bioaccumulable metalloid that originates from both anthropogenic and natural sources and causes detrimental effects in humans and wildlife [31]. Arsenic in the Odiel and Tinto estuary originates from historic and current mining activity [32,33,34]. An estimated 12 t yr^-1^ and 23 t yr^-1^ of As is transported by the Tinto and Odiel rivers (respectively) into the Atlantic Ocean [35]. Previous studies have found high levels of inorganic arsenic in sediments (85–610 ppm) in the study area [36], exceeding the ERM (Effects Range Median) for marine and estuarine sediments (70 ppm [37]), and also the Canadian sediment quality guideline (CSQG) value for the protection of aquatic life (7.2 ppm [38]).

This study was designed to test the individual and combined effects of infection by cestode parasites and a 4°C temperature change on the sensitivity of *Artemia* to acute As exposure. We conducted toxicity tests to compare As median lethal concentrations at 24 h (24-h LC_50_) for infected and uninfected individuals on two separate dates one month apart, in order to test the effect of different parasite assemblages on arsenic sensitivity. Since cestode parasites in *A*. *parthenogenetica* have a strong seasonal pattern [29], and different parasite species induce different physiological effects in their host, we chose to consider the effects of different parasite compositions (i.e dates). We also evaluated the effect of parasites on the antioxidant defense mechanisms of *Artemia*, in order to measure the capacity of infected animals for detoxification of reactive oxygen species caused by factors such as pollution or climate change. We included the activity of four important enzymes: superoxide dismutase (SOD) responsible for the detoxification of the highly reactive oxygen species superoxide anions; catalase (CAT) which catalyses the decomposition of hydrogen peroxide in 0_2_ and H_2_0; glutathione peroxidase (GPX) which acts as a scavenger for high concentrations of hydrogen peroxide; and glutathione reductase (GR) implicated in the reduction of glutathione disulphide to the sulphydryl form glutathione, which is a critical molecule in combating oxidative stress. In order to evaluate the efficacy of the above enzymatic mechanisms for control of reactive oxygen species, we also measured the levels of thiobarbituric acid reactive substances (TBARS), which indicates the extent of lipid peroxidation as a consequence of oxidative damage. Finally, we quantified the carotenoid-rich lipid droplets of infected and uninfected individuals. These droplets serve as intracellular lipid storage but also play a protective role in protecting cells against oxidative stress.

## Results

### Acute toxicity tests (median lethal concentrations, 24-h LC_50_)

#### Infection characteristics between sampling dates

**Fig 1:**
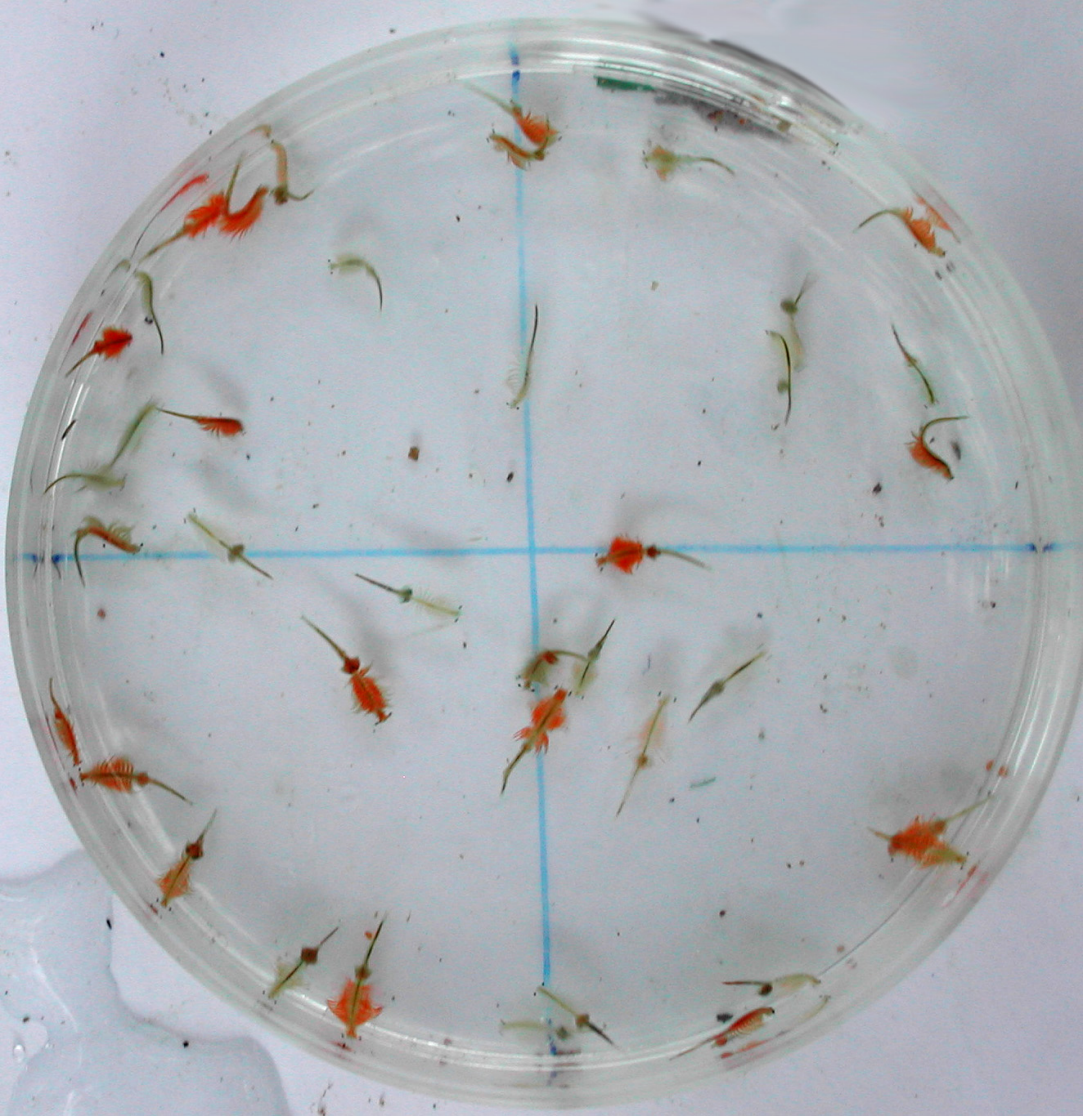
Infected and uninfected *Artemia*. Infected (red) and uninfected (transparent) *Artemia parthenogenetica* from Odiel and Tinto estuary.

In order to test the effect of different parasite assemblage on the response of *Artemia* to As, two experiments were conducted in different months (April and May 2014). Infected and uninfected individuals were separated on the basis of their colour ([39], Fig 1), and their parasitic status was confirmed after the experiment.

Infection indices (prevalence and abundance) for six of nine cestode species recorded in the *infected group* varied significantly between the two experiments (Table 1).

**Table 1:** Infection indexes of *Artemia* at different dates in 2014.

Parasite species	Infection descriptor	14-April	15-May	Z test	M-W U test
*Flamingolepis liguloides*	P (%)	68.00 ± 2.6	89.8 ± 1.2	8.52 *	
	MA	1.20 ± 0.065	1.50 ± 0.041		128423.5 **
*Flamingolepis flamingo*	P (%)	6.00 ± 1.3	0.90 ± 0.4	21.57 *	
	MA	0.058 ± 0.013	0.015 ± 0.0083		103318.5 **
*Confluaria podicipina*	P (%)	65.20 ± 2.6	0.00	23.29 *	
	MA	0.74 ± 0.036	-		37835 **
*Wardium stellorae*	P (%)	3.00 ± 1.00	1.70 ± 0.5	13.22 *	
	MA	0.033 ± 0.0099	0.017 ± 0.005		106766
*Anomotaenia microphallos*	P (%)	1.00 ± 0.5	7.00 ± 1.00	1.52	
	MA	0.0091 ±0.0052	0.071 ± 0.011		115011 **
*Anomotaenia tringae*	P (%)	2.1 ± 0.8	12.30 ± 1.3	5.15 *	
	MA	0.024 ± 0.0085	0.12 ± 0.013		119138 **
*Eurycestus avoceti*	P (%)	13.3 ± 1.9	5.20 ± 0.9	4.19 *	
	MA	0.13 ± 0.019	0.49 ± 0.0084		99374 **
*Fimbriarioides tadornae*	P (%)	0	0.20 ± 0.2	-0.11	
	MA	-	0.0015 ± 0.0015		108735
*Gynandrotaenia stammeri*	P (%)	0	0.20 ± 0.2	-0.11	
	MA	-	0.0015 ± 0.0015		108735
TOTAL PARASITES	P (%)	98.00 ± 0.7	98.00 ± 0.5	-0.81	
	MA	2.2 ± 0.012	1.78 ± 0.0079		8437739**
Species Richness		1.61 ± 0.03	1.24 ± 0.02		71953**

Prevalence (P%), Mean Abundance (MA ± SE) and Species Richness (± SE) of cestodes in *Artemia* samples assigned to infected experimental treatments based on coloration. A Z test was used to compare Prevalence between dates and a Mann-Whitney U test to compare mean abundance and species richness between dates.

* P < 0.01

** P < 0.001

**Fig 2:**
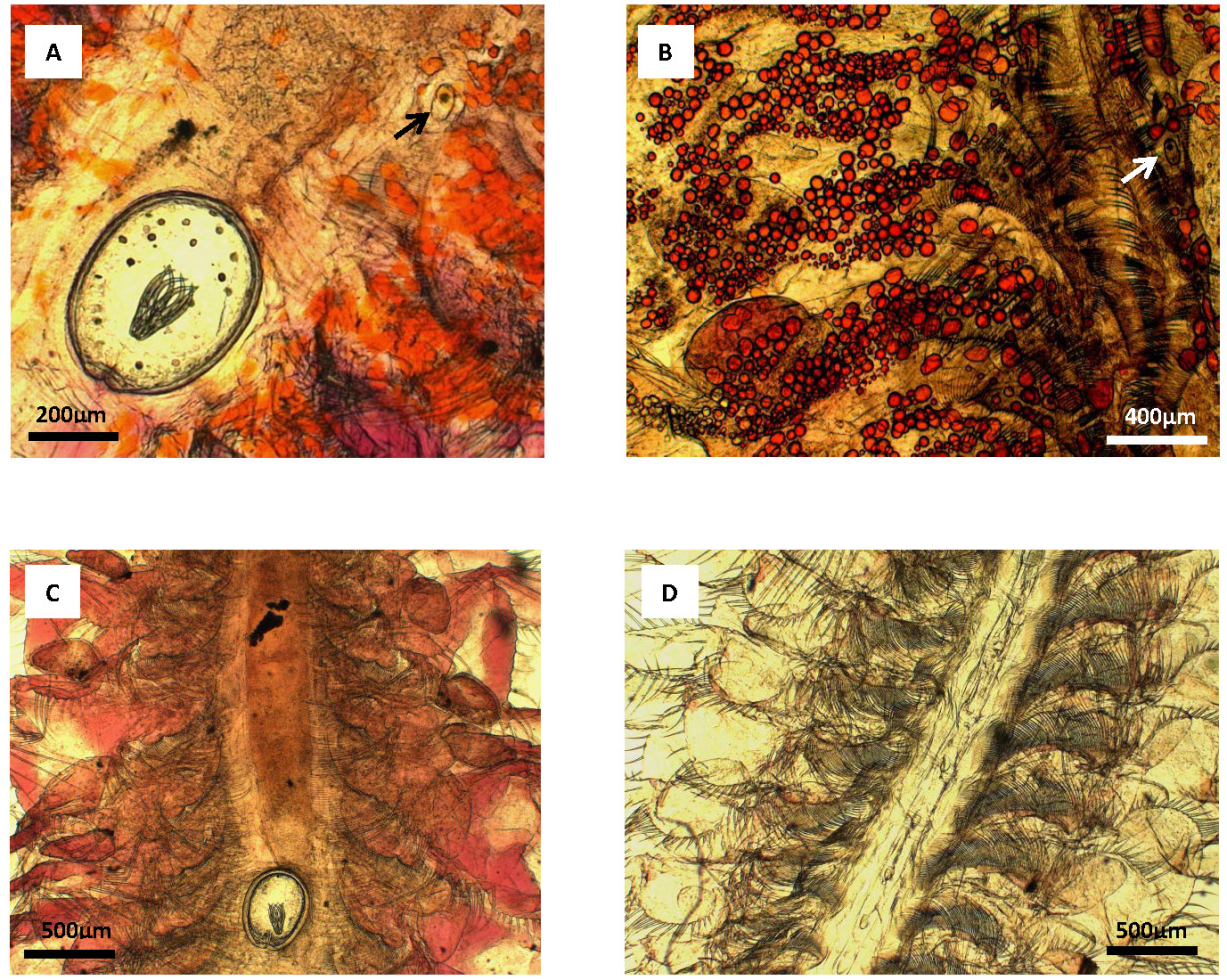
Lipid droplets with carotenoid pigments, and hemoglobins in *Artemia* infected by *Flamingolepis liguloides* and/or *Confluaria podicipina*. *Artemia parthenogenetica* infected by cysticercoids of *Confluaria podicipina* (indicated by arrows) and *Flamingolepis liguloides* in mixed infection (A), simple infection by *C*. *podicipina* (B), simple infection by *F*. *liguloides* (C) and uninfected *Artemia* (D). Lipid droplets with carotenoid pigments are visible as orange colouration in A and B (much more pronounced in B); hemoglobins are visible as pink colouration in A and C.

Infected individuals in April were dominated by *Flamingolepis liguloides* (68%) and *Confluaria podicipina* (65%), with 36% of individuals being infected by both these parasites (Fig 2).

In contrast, infected individuals in May were dominated by *F*. *liguloides* (90%) with no *C*. *podicipina*. Other minority cestodes (prevalences of 0.2–14%) present in one or both of the experiments were *Flamingolepis flamingo* and *Gynandrotaenia stammeri* (with flamingos as final hosts), *Eurycestus avoceti*, *Anomotaenia microphallos* and *A*. *tringae* (shorebirds), *Fimbriarioides tadornae* (shelducks) and *Wardium stellorae* (gulls). Total parasite loads were significantly higher in April, as was the number of cestode species present per individual *Artemia* (Table 1). The proportion of infected *Artemia* with only a single cysticercoid increased from 35.9% in April to 52.2% in May. Likewise, the proportion of infected *Artemia* infected by only a single cestode species increased from 46.3% in April to 77.1% in May.

#### Median lethal concentration—LC_50_

**Fig 3:**
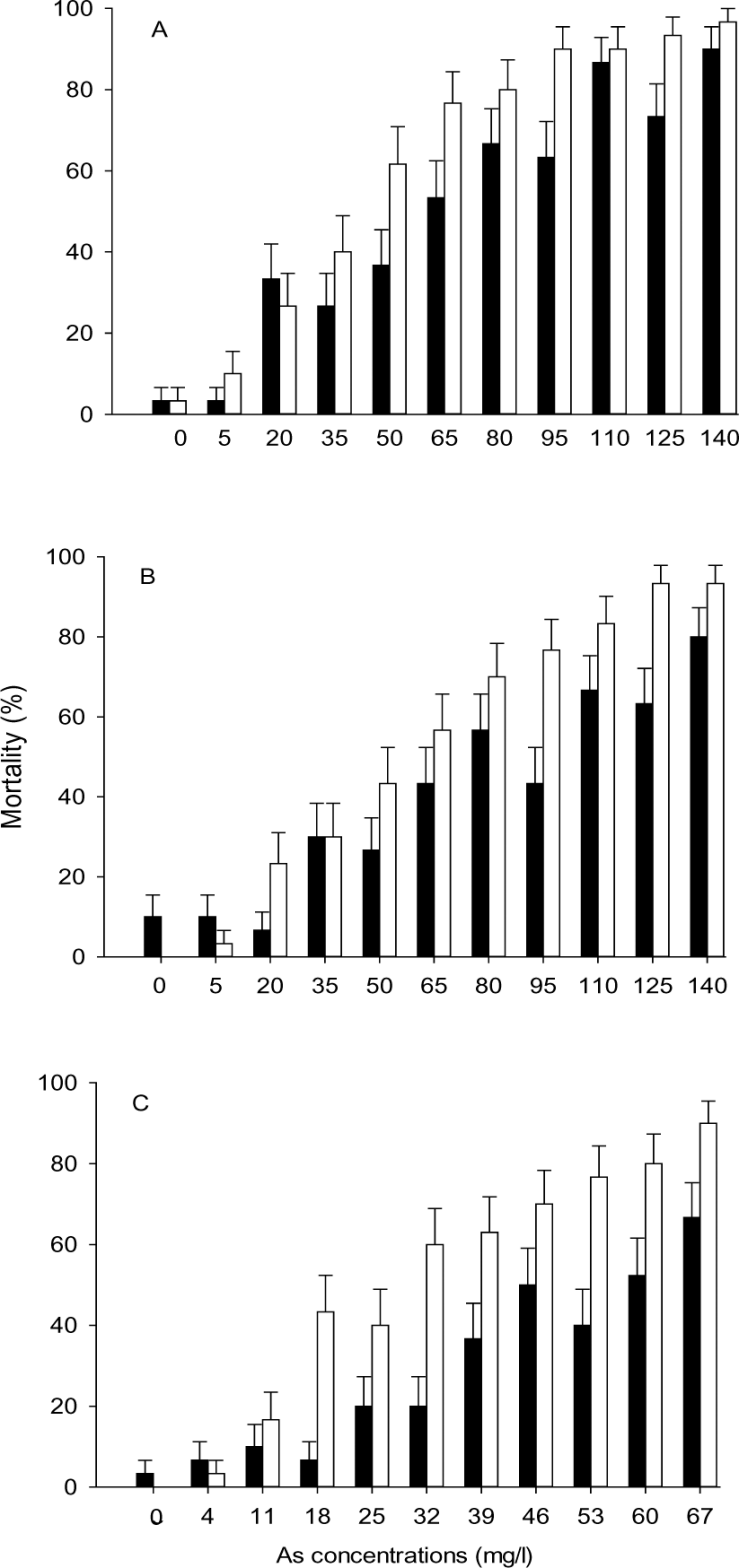
Mortality of *Artemia* exposed to As. Mortality (% mean ± SE) of *Artemia* after exposure to different As concentrations (mg/l) during 24 h at 25°C (A, B) and 29°C (C), at different dates with different parasite assemblages (A: April; B, C: May). Black bars = infected individuals (n = 330, n = 330, n = 328 for A, B and C respectively); white bars = uninfected individuals (n = 328, n = 330, n = 329). Note the changes in scale on the X axis.

We conducted acute toxicity tests (median lethal concentration, 24-h LC_50_) in infected and uninfected individuals at a single temperature (25°C in April) or at two temperature conditions (25 and 29°C in May). In April, when *Artemia* starts the first generation of the year, there were not enough individuals to conduct the experiment at 29°C. However it was a good moment to conduct the experiments because at the beginning of the season a high proportion of *Artemia* are infected by only one parasite species, making results easier to interpret (later in the year, mixed infections are rather common, with of up to 8 cestode species coexisting in a single host [29]). Mortality of infected *A*. *parthenogenetica* was consistently lower than that of uninfected *Artemia* under different As concentrations (Fig 3).

**Fig 4:**
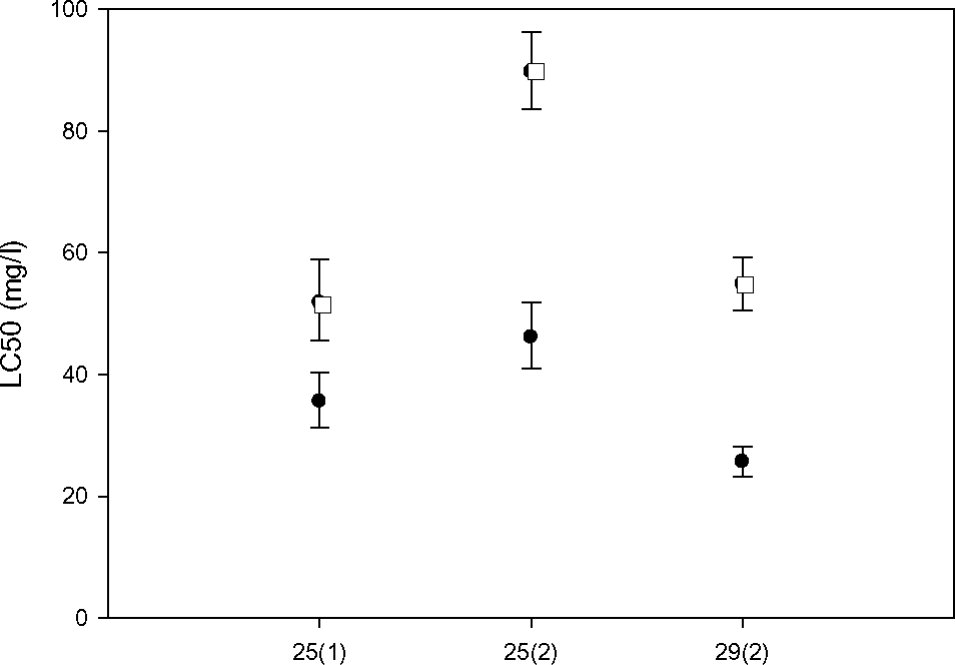
Median lethal concentrations at 24 h (LC_50_) of As for infected and uninfected *Artemia*. LC_50_ of infected (empty squares) and uninfected (solid circles) *Artemia parthenogenetica* at different dates with different parasite assemblages (1 = April, dominance by *F*. *liguloides* and *C*. *podicipina*; 2 = May, dominance by *F*. *liguloides* with lower total infection intensity) and temperatures. Error bars correspond to 95% confidence limits.

LC_50_ was significantly higher in infected individuals for both dates and parasite assemblages, and at both temperatures, as indicated by clear separation of confidence intervals [40] (Fig 4).

However, whilst the 24h LC_50_ of the uninfected individuals at 25°C did not change between the two dates, that for infected individuals was significantly higher in May (when *F*. *liguloides* infection was more dominant and total parasite loads were lower) compared with the experiment conducted in April (when both *F*. *liguloides* and *C*. *podicipina* were highly prevalent) (Fig 4). An increase in temperature decreased As tolerance in both infected and uninfected *Artemia* (Fig 4).

In a Generalized Linear Model (GLZ), mortality rate (i.e., the number of *Artemia* that died) was significantly influenced by temperature (p = 0.015), date (p = 0.007), parasitic status (p < 0.0001) and As concentration (p < 0.0001) (Table 2). The interactions “parasitic status x temperature” and “As concentration x temperature” were also significant (p < 0.01 and p < 0.0001, respectively).

**Table 2:** GLZ on mortality rate of *Artemia* exposed to Arsenic.

	Level of Effect	Estimate	Standard Error	Wald Stat.	p
Intercept		0.368227	0.078794	21.8398	0.000003
Temperature	25°C	0.191934	0.079357	5.8496	0.01558
Date	April	0.086186	0.032191	7.1681	0.007421
Infection status	Infected	-0.20015	0.031315	40.8507	0.000000
As concentration		0.022135	0.001391	253.217	0.000000
Temp*Infection status		0.080426	0.031315	6.596	0.010221
Temp*As concentration		-0.009019	0.001391	42.0372	0.000000
Scale parameter		0.825806	0.000000		

Generalized linear model of *Artemia parthenogenetica* mortality with number of dead individuals per replicate (n = 10 individuals, with three replicates per As dose) as dependent variable with a Poisson error function and log link function. Temperature 29°C, date May and uninfected status are aliased reference categories.

### Oxidative stress and lipid droplet quantification in infected and uninfected *Artemia*

It was impossible to analyse oxidative stress in individuals used in acute toxicity tests because of the need to crush the specimens in order to check for parasites, so we conducted a separate sampling in May 2015. Parasite prevalence has a seasonal pattern [29], so in order to have a similar parasite assemblage, samples were collected in the same calendar month as those used in toxicity tests and from the same ponds of medium-high salinity (140–220 g/l). One subsample was used for oxidative stress analysis and another to characterize parasite assemblage and to quantify lipid droplets. Infected and uninfected individuals were recognised on the basis of their colour [392]. Table 3 shows the infection index of infected individuals. As expected, infection was dominated by *F*. *liguloides*, and its prevalence did not differ from the sampling of May 2014 (Mann-Whitney U test, U = 13158, P = 0.585). Prevalence and abundance of minority cestode species varied among dates.

**Table 3:** Infection indices of infected *Artemia* in 2015.

	P (%)	MA
*Flamingolepis liguloides*	81.7	0.92 ± 0.076
*Flamingolepis flamingo*	11.7	0.13 ± 0.023
*Confluaria podicipina*	3.3	0.033 ± 0.023
*Euricestus avoceti*	3.3	0.033 ± 0.023

Prevalence (P) and mean abundance (MA ± SE) of different cestode species infecting *Artemia parthenogenetica* (n = 60) from Odiel salt ponds in May 2015.

**Fig 5:**
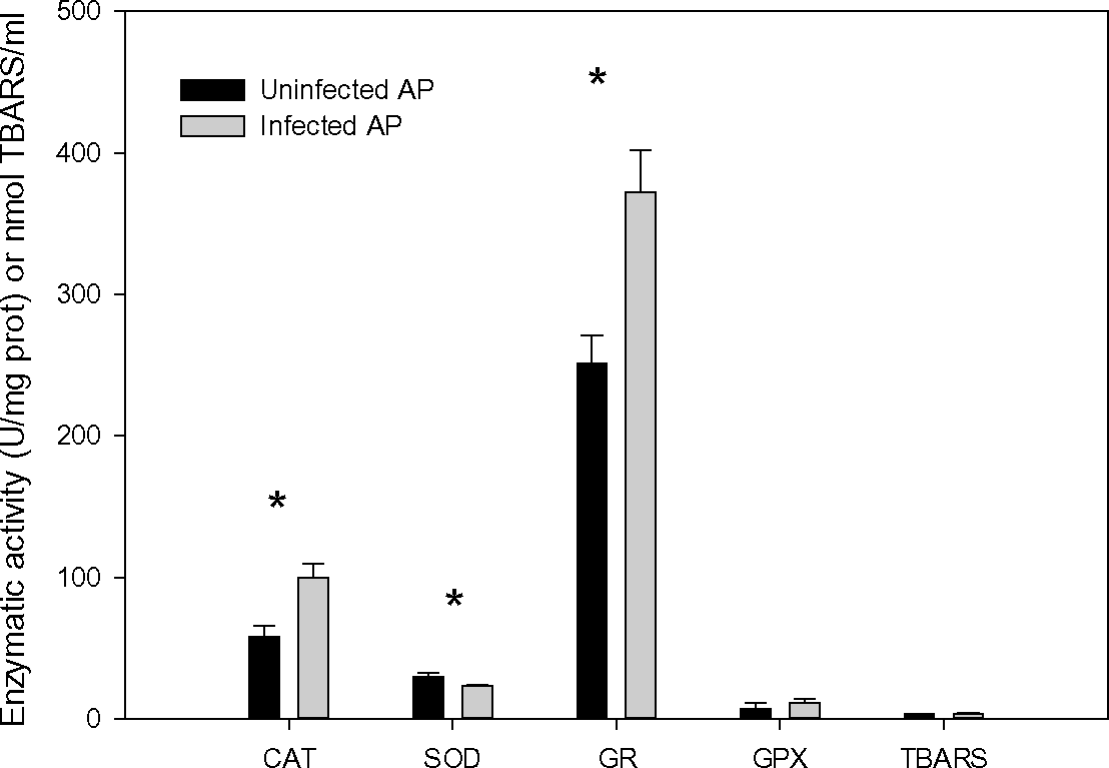
Oxidative stress biomarkers in infected and uninfected *Artemia*. Comparison of oxidative stress biomarkers (mean ± SE) of infected and uninfected *A*. *parthenogenetica* from Odiel in May 2015. Results are presented in U/mg protein (catalase, CAT; superoxide dismutase, SOD; Glutathione reductase, GR; and glutathione peroxidase, GPX) or nmol TBARS/ml. *Statistical differences between infected and uninfected individuals with a P value < 0.05.

The analysis of oxidative stress in infected and uninfected *Artemia* revealed the association of parasites with strong changes in the antioxidant capacity of the host. Infected individuals showed significantly higher levels of CAT (Fig 5, t = -2.892, P = 0.02) and GR activities (Fig 5, t = -2.881, P = 0.002), whereas SOD was higher in uninfected individuals (t = 2.739, P = 0.03). Conversely, parasites had a negligible effect on GPX (t = -0.814, P = 0.447) and TBARS (t = -1.781, P = 0.125).

**Fig 6:**
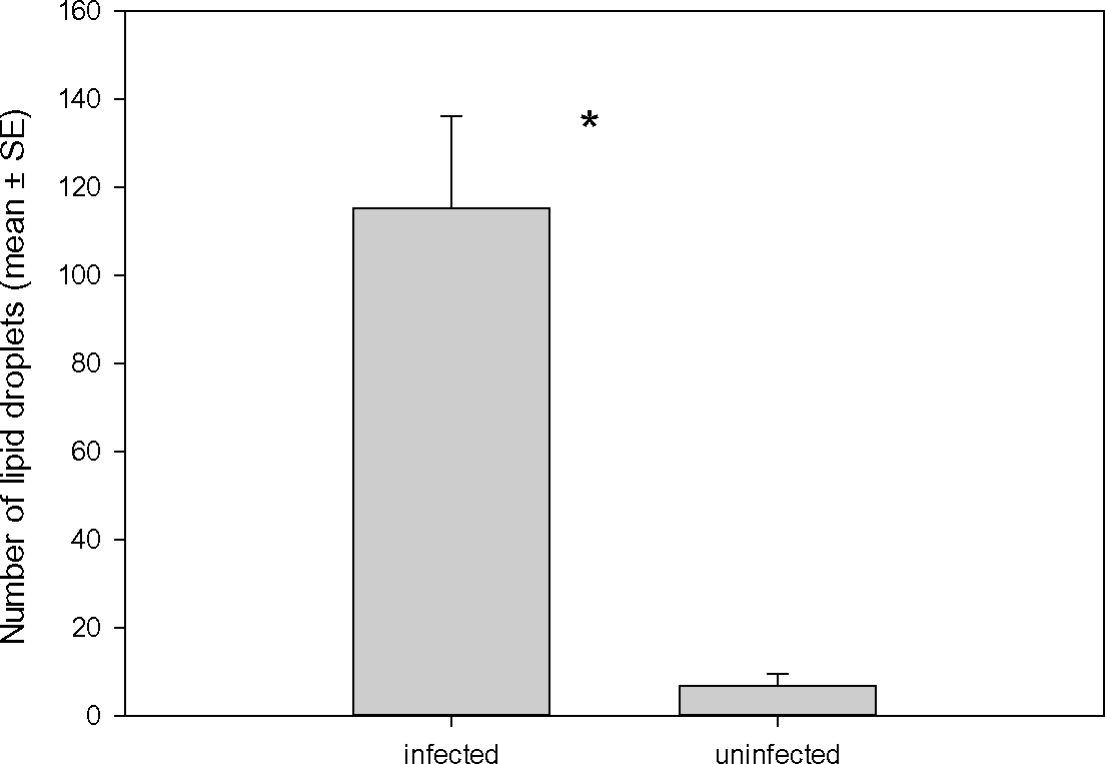
Lipid droplets in infected and uninfected *Artemia*. Number of lipid droplets (mean ± SE) in infected and uninfected *Artemia parthenogenetica* from Odiel in May 2015. *Significant differences between infected and uninfected individuals with a P value < 0.001.

We also quantified lipid droplets, which are readily visible throughout the body and appendices of adult *Artemia* (Fig 2B). Lipid volume estimates are strongly correlated with biochemical measurements of lipids [41], and are highly related with the ability of organisms to protect themselves against pollutants [42]. Infection with cestodes was associated with increased number of lipid droplets (Mann-Whitney U test, U = 45.5, P < 0.001, n = 20; Fig 6).

## Discussion

### Do cestode parasites affect As toxicity in *Artemia*?

This study tested the effect of parasites on *Artemia* sensitivity to As, and explored the physiological crosstalk between the parasite and the host, measuring oxidative stress and lipid content in infected and uninfected *Artemia*. Our study provides the first empirical evidence that parasites can increase resistance to metal or metalloid pollution, rather than decrease it. It is also the first study to consider the influence of temperature change on parasite-pollutant interactions. In three separate acute toxicity experiments, *Artemia* infected with cestodes consistently showed lower sensitivity to As than uninfected individuals. The higher sensitivity of infected *Artemia* in April suggests that multiple infections may reduce the benefits of cestode infection to host resistance.

Our results contradict the pre-existing view that pollution and parasites are stressors that both have negative effects on the health of free living organisms. This view was based on previous field and laboratory investigations (including chronic and acute exposure to a wide variety of toxicants, in vertebrates and invertebrates, intermediate and definitive hosts, and in several groups of parasites [43,44,45].

The results of oxidative stress analysis provide a mechanistic explanation for our findings. Infected individuals exhibited much higher levels of CAT and GR, reflecting a superior ability to combat the effects of exposure to pollutants with oxidative potential, such as As. The particularly high levels of CAT in infected individuals (nearly double that of uninfected *Artemia*) is related to the increased levels of hemoglobins in *F*. *liguloides*-infected *Artemia* compared with uninfected individuals (Fig 2C and 2D). CAT is a haematin protein complex with four porphyrin heme groups that allow the enzyme to react with hydrogen peroxide. There is a close linear relationship between CAT activity and hemoglobin concentration in human blood [46]. CAT also has one of the highest rates of all enzymes; one CAT molecule is able to catalyse the conversion of 5 million molecules of hydrogen peroxide per second to water and oxygen. Thus, even if the level of SOD was lower in infected individuals, the control mechanisms via CAT and GR seem to be sufficient to avoid the establishment of an oxidative stress condition, as indicated by the lack of changes in TBARS between infected and uninfected individuals. TBARS is a by-product of lipid peroxidation, so this result indicates that parasites are not inducing damage by oxidative stress. Our results conflict with what most studies have shown up to now (but see for example Marcogliese et al 2005 [6]). Infection by parasites and pathogens of a wide range of taxa are generally associated with the inhibition or weakening of the host antioxidant system, and the concomitant increase in TBARS [47,48,49].

This enhancement of the anti-oxidative defense mechanisms is probably connected with the trophic transmission mode of cestodes that infect *Artemia*, which means they require “healthy” hosts in order to increase transmission success (through predation by the definitive host). Decrease in antioxidant status enhances short-term survival prospects [50], so potentiating it may be part of the transmission strategy of the parasite to increase longevity and probability of transmission (as in the “parasite manipulation hypothesis” [51]).

We also found that there were more lipid droplets in infected individuals, which is consistent with previous work indicating that *F*. *liguloides* increases total lipid levels [52] and that *C*. *podicipina* increases triglyceride levels in *Artemia* [26] (Fig 2B). Lipids have a high heavy metal binding capacity, and lipid content has a significant effect on the accumulation of As in other organisms [53]. Neutral lipids such as those in lipid droplets can protect organisms against pollutants, sequestering them away from sensitive target sites [54,55]—a principle known as *survival of the fattest* [41]. Although many studies support this principle, none have addressed parasite-mediated effects. The only previous study to suggest that parasites can increase host survival under polluted conditions through a lipid-related effect was on the freshwater clam *Pisidium amnicum* [56]. Clams infected by digenean trematode larvae are less sensitive to pentachlorophenol, perhaps because the high lipid content of the parasite changes the internal distribution of the toxicant. Pentachlorophenol is moderately lipophilic so is expected to accumulate in adipose tissue, whereas the target sites for toxicity are the mitochondria [56]. In parasitized *Artemia*, additional lipids accumulate in the host, not in the parasite as in the case of *Pisidium*.

Lipid droplets in infected *Artemia* are associated with carotenoids (see Fig 2B) and this, together with hemoglobins induced by parasites, largely explains the colour change that allowed us to separate infected individuals with the naked eye (Fig 1). Both *F*. *liguloides* and *C*. *podicipina* increase the concentration of carotenoids in infected *Artemia* [52]. In contrast, carotenoid concentrations in other animals are often negatively correlated with parasite load [57] and with pollutants [58]. Carotenoids are potent lipid-soluble antioxidants [59] and are able to inhibit lipid peroxidation in liposomes [60]. The accumulation of carotenoids in infected *Artemia* is also considered a parasite strategy to increase the probability of being predated by birds (the final host) by increasing visibility [61] and enhancing nutritive value [26]. Carotenoids provide protection against oxidative stress in many free living organisms [62,63,64] so they may also increase longevity in infected *Artemia*. Given that oxidative stress is a common marker of toxicity, not only for As in plants, invertebrates and vertebrates [65,66,67] but also for heavy metals (e.g. lead, cadmium and mercury [68]), cestode parasites may protect *Artemia* against a broader range of pollutants.

Unlike carotenoids, a positive effect of parasites on host lipid content is common in nature, e.g. in acanthocephalans infecting gammarids [69] or trematodes infecting bivalves [70]. Therefore, our finding regarding increased resistance to As in the presence of parasites may not be an isolated case, and more studies are needed to evaluate how frequently this occurs in nature.

The differences in sensitivity to As observed in infected *Artemia* collected on different dates have several possible explanations, including a negative effect of the generally higher infection levels, or a higher pathogenicity of *C*. *podicipina* which was absent in May. Alternatively, it could be related to seasonal changes in the ages of the parasites or their hosts, or changes in the lipid or carotenoid levels in the host. Increased heavy metal accumulation with age in cysticercoids has been shown in other cestodes [71,72]. Previous studies of the interactions between parasites and pollutants on toxicity have focused on individual parasite species (but see Gismondi et al. [7]). In nature, co-infections of different parasites are extremely common, and our study illustrates the need to consider the effects of co-infections in environmental parasitology.

### Effect of temperature on As toxicity

The toxicity of As is highly dependent on its bioavailability, which in turn is dependent on its chemical form and the capacity to be released from environmental matrices [73]. In marine environments, As occurs predominantly as the inorganic forms arsenate (As(V)), and arsenite (As(III)), and is significantly more bioavailable from seawater and porewater than from sediments [74]. Moreover, temperature can strongly affect the chemical behaviour of pollutants and their bioavailability [75,76,77], but also the physiology of aquatic organisms. There are many studies of the effect of temperature on heavy metal toxicity, but we are not aware of any that integrate the influence of parasites. A temperature rise of 4°C caused a significant decrease in the LC_50_ for both infected and uninfected *Artemia*. The decrease in dissolved oxygen in hypersaline waters with increasing temperature coincides with higher respiratory demands in *Artemia*. Water permeability and drinking in *Artemia* increase markedly with temperature [78], hence uptake of pollutants will also increase. Indeed, copper uptake in *Artemia* increases with temperature owing to increased activity and diffusion rate [79]. Differences in As toxicity in fish have also been associated with higher uptake at higher temperatures [80].

### Conclusions

Parasites and pathogens are conventionally considered as detrimental for a host, but they can also have positive impacts with consequences for non-host species and even the whole ecosystem [81]. We provide evidence that parasites can also benefit their hosts by increasing resistance to pollutants in contaminated environments. Infection by parasites was associated with an improved antioxidant defense system (CAT and GR) without oxidative damage, as confirmed by unaffected values of TBARS. Parasites also increased the number of lipid droplets in infected individuals, which is a common phenomenon in intermediate hosts manipulated by parasites, so more studies in other host-parasite systems are needed to evaluate the wider relevance of our findings.

Our results provide an important advance in our understanding of host-parasite interactions and underline the importance of considering interactions between parasites, pollutants and temperature in combination, particularly given expected climate change and the likelihood that toxicity will increase with temperature.

## Material and Methods

### Short-term toxicity of As (LC_50_)

Naturally infected and uninfected adults of *A*. *parthenogenetica* were collected with a plankton net (0.5 mm) within the Odiel saltpan complex (see Sánchez *et al*. [21] for details of the study area).

Sampling was carried out on two dates (14^th^ of April and 15^th^ of May 2014) at which the relative abundance of different cestode parasites changed considerably. On the 14^th^ of April, *Artemia* were collected from three ponds with salinities of 140, 150 and 190 g/l (measured with a refractometer). On the 15^th^ of May, samples were collected from another pond with a salinity of 200 g/l. These four ponds were all within the same stage of the solar evaporation process, were hydrologically interconnected, and had similar sediment type, depth, and invertebrate and bird communities [21]. These ponds were selected on the basis of the abundance of live *Artemia*. *Artemia* were transported to the laboratory in 25 litre containers. Infected and uninfected *Artemia* individuals of a similar size were then selected on the basis of their colour (Fig 1); infected individuals are visually recognisable by the bright red colouration induced by the cestodes, whereas uninfected individuals are practically transparent [39]. Similar size (as a proxy of age) was selected since this may influence As concentrations or sensitivity [82, 83]. There is no difference in growth rates between infected and uninfected shrimp [52]. Of the individuals visually allocated to the *infected group* prior to experiments, 98% were truly infected when inspected afterwards (n = 989). Among individuals allocated to the *uninfected group*, 98% were truly uninfected (n = 988).

Toxicity experiments were conducted after 24 h of acclimation of the *Artemia* at 100 g/l salinity (artificial salt mixture of *Instant Ocean* dissolved in distilled water). Two experiments were carried out. The first (with *Artemia* collected on 14^th^ of April) was conducted at 25°C (the mean annual temperature in the Odiel salt pans [21]). The second (*Artemia* collected on 15^th^ of May) was conducted at both 25 and 29°C. During summer months, *Artemia* are often exposed to temperatures ≥ 29°C [21, 22] and the frequency of these events is expected to increase in future.

Median lethal concentration (LC_50_) was used to quantify As toxicity in infected and uninfected adult *Artemia*. Arsenic, as reagent-grade sodium arsenate, NaAsO (CAS No. 10048-95-0) was used to prepare a concentrated stock solution. The study design concentrations were prepared by mixing different proportions of the stock solution and saltwater (Instant Ocean prepared with MilliQ). The saltwater used for the dilutions was prepared within 24 h of the start of the experiment and As added one hour before conducting the experiment (to prevent oxygen depletion). Ten concentrations of As between 5 and 140 mg/l were used for the experiments at 25°C (0, 5, 20, 35, 50, 65, 85, 95, 110, 125, 140 mg/l), and ten between 4 and 67 mg/l were used for the experiment at 29°C (0, 4, 11, 18, 25, 32, 39, 46, 53, 60, 67 mg/l) in order to estimate the LC_50_. Experimental concentrations were adjusted to the observed mortality (higher mortality at 29°C implied lower tested concentrations). These concentrations were selected after preliminary tests. Three replicates were used per concentration, with each replicate made up of a group of 10 individuals. Infected (red) and uninfected (transparent) individuals were transferred to 25 ml beakers (10 individuals per beaker) and placed in climatic chambers at the chosen temperature, with a 12:12 photoperiod and without food. After a 24 h exposure period, dead individuals (considered to be those with no movement of the appendages observed within 10 seconds) were counted and all (alive or dead) individuals were mounted on slides to confirm parasitic status under the microscope. After observations of the cysticercoids (cestode larval stage in the intermediate host) *in situ*, each infected specimen was gently pressed under the coverslip. If the identification of the cysticercoids recorded was not possible at this stage, whole infected brine shrimps or isolated cysticercoids were prepared as permanent mounts in Berlese’s medium in order to facilitate observations on the morphology of rostellar hooks. Cysticercoids were identified following Georgiev *et al*. [30] and Vasileva *et al*. [84]. Prevalence (P) and mean abundance (MA) were calculated separately for the “*infected group*” on both dates.

### Oxidative stress analysis and lipid droplet quantification

*Artemia* were sampled in May 2015 from Odiel salt ponds of intermediate-high salinity. Brine shrimps were transported to the laboratory and placed in artificial sea water (Instant Ocean, 100 g/l) before the experiment. A subsample was used to characterize the exact parasite composition (n = 60 infected individuals) and quantify the number of lipid droplets (n = 20 infected and 20 uninfected *Artemia*). The numbers of cysticercoids, prevalence, mean abundance and mean intensity (see Bush et al. 1997 [85] for definitions) were calculated for each cestode species. The number of lipid droplets was estimated according to Wurtsbaugh & Gliwicz 2001 [86]. We quantified lipid levels by inspecting individuals at 30x magnification and counting the number of lipid droplets along the right side of the 5th and 6th segments of the body.

The rest of the specimens were acclimated during 24h to the experimental salinity with continuous aeration and fed *ad libitum* with lyophilized *Tetraselmis chuii* algae. The toxic concentrations of 4.69 mg/l As was selected on the basis of preliminary LC_50_ tests (the lowest concentration at which mortality was detected). Infected and uninfected *A*. *parthenogenetica* of the same size range were allocated to 1L glass vials (100 individuals per vial) with 600 ml of experimental solution (either control (no As) or 4.69 mg/L As) during 24h at 25°C (12:12 photoperiod) without food.

After 24h exposure, individuals were gently washed in distilled water and, after removing excess water, stored at ‒80°C until biochemical analysis. All operations were performed at 4°C to prevent enzyme or tissue degradation. We performed the biochemical analysis on pools of 20 individuals per treatment. Number of replicates varied from 1 to 12 depending on *Artemia* availability. The different biomarkers were determined in the whole soft tissues after homogenization and centrifugation. Tissues were homogenized with an electrical homogenator (Miccra D-1 Art Moderne Labor Technik) in cool homogenization buffer (Tris-HCl 100 mM, EDTA 0.1 mM, Triton X-100 0.1%, pH7.8) using 200 μl of buffer per sample (20 individuals). The sample was centrifuged at 14,000 rpm at 4°C for 30 minutes and supernatant stored at −80°C until enzymatic determination.

We quantified five parameters as a proxy for oxidative status of *Artemia*: activity of four enzymes (catalase CAT, superoxide dismutase SOD, glutathione peroxidase GPx and glutathione reductase GR) and lipid peroxidation levels (thiobarbituric acid reactive substances TBARS). Total protein content in the supernatant fluid was determined following a standard Bradford’s procedure [87]. Enzyme activity was determined colorimetrically. Concentration of each indicator was estimated following the specific procedures below.

#### Catalase (CAT) activity

Catalase activity was analyzed according to the method of Cohen *et al*. (1970) [88] by monitoring the enzyme-catalyzed decomposition of hydrogen peroxide using potassium permanganate. Catalase enzyme prevents the accumulation of H_2_O_2_ by converting it to H_2_O and O_2_ as follows:.

H2O2 → 2H2O + O2

Potassium permanganate (KMnO4) is a powerful oxidizing agent that decomposes hydrogen peroxide through the following reaction:

2 KMnO4 + 5 H2O2 + 3H2SO4 → 2 MnSO4 + 8 H2O + 5O2 + 3K2SO4

This produces a red end product. Absorbance at a wavelength of 480 nm is read five minutes after KMnO_4_ is added to the samples. Catalase activity is quantified by extrapolating in a standard curve of commercial catalase (SIGMA-60634), and is expressed as U/mg of total proteins.

#### Superoxide dismutase (SOD) activity

Superoxide dismutase catalyses the dismutation of superoxide radicals to oxygen and hydrogen. SOD was estimated using the xanthine-oxidase cytochrome c method as described by McCord and Fridovich (1969) [89]. The inhibition of cytochrome C was followed spectrophotometrically at 550 nm. One unit of SOD activity is defined as the amount of enzyme providing 50% inhibition of the control rate of cytochrome c reduction at 25°C. SOD activity is expressed as U/mg of total proteins.

#### Glutathione peroxidase (GPx) activity

Selenium-dependent glutathione peroxidase (Se-GPx) exerts its protective role by reducing organic peroxides and hydrogen peroxide to alcohols and water, respectively, using glutathione (GSH) as a hydrogen donor. When GSH is oxidized to GSSG it loses its protective function. The GPx activity was determined by estimating nicotinamide adenine dinucleotide phosphate (NADPH) oxidation following the method of Carmagnol et al. (1983) [90], which is a modification of the Paglia and Valentine (1967) method [91]. During the assay, the GSSG is continually reduced by an excess of glutathione reductase (GR), producing a constant level of reduced glutathione (GSH). Production of GSH from oxidised glutathione requires NADPH. Therefore, the activity of GPx is measured by the reduction in the absorbance at 340nm due to the oxidation of NADPH, and is expressed as mU/mg of total proteins.

#### Glutathione reductase (GR)

Glutathione reductase (GR) plays an essential role in protection against oxidative damage by catalysing the reduction of glutathione disulphide (GSSG) in a reaction dependent on NADPH. The activity of GR was determined, as for GPx, by measuring the decrease in absorbance at 340 nm due to the oxidation of NADPH, which acts in the reduction of GSSG to GSH, following the method of Cribb et al. 1989 [92]. GR activity is expressed as mU/mg of total proteins.

#### Thiobarbituric acid reactive substance (TBARS)

Lipid peroxidation was estimated by the formation of thiobarbituric acid reactive substances (TBARS) following Buege and Aust (1978) [93]. Lipid peroxidation produces malondialdehyde (MDA), which is formed from the breakdown of polyunsaturated fatty acids. MDA reacts with thiobarbituric acid, producing a red species which is optically measured at 535nm. Lower detection limit for TBARS was 0.1 μM, and the average coefficient of variation for sample duplicates was 3.54%. TBARS was measured in mmol MDA/mg.

All biochemical analyses were performed at the Ecophysiology Laboratory of the EBD-CSIC in a Victor 3 multiplate reader (PerkinElmer, Massachusetts, USA).

### Statistical analysis

The median acute lethal concentration (LC_50_) and 95% confidence limits were estimated and compared between infected and uninfected individuals, different temperatures and dates using Trimmed Spearman-Karber (TSK) analysis for lethal tests [94]. The criterion of *non-overlapping 95% confidence limits* was used to determine a significant difference (p < 0.05) between LC_50_ values [40].

To test the effect of date, temperature, parasitic status and As concentration on mortality we performed GLZ with a Poisson error distribution, log link function and correction for overdispersion. Date, temperature and parasitic status were included as categorical factors, and As concentration as a continuous variable. Stepwise backwards removal was used to obtain a final model containing only significant factors.

Differences in prevalence of different cestode species between the two sampling dates were evaluated with Z-tests. Comparisons of cestode abundance were performed with Mann-Whitney U tests. We also used Mann-Whitney tests to compare enzymatic activity and lipid peroxidation, as well as lipid droplets between infected and uninfected individuals. Statistica 12 software for Windows was used for all statistical analyses [95].
